# Monitoring of Antiretroviral Therapy and Mortality in HIV Programmes in Malawi, South Africa and Zambia: Mathematical Modelling Study

**DOI:** 10.1371/journal.pone.0057611

**Published:** 2013-02-28

**Authors:** Janne Estill, Matthias Egger, Leigh F. Johnson, Thomas Gsponer, Gilles Wandeler, Mary-Ann Davies, Andrew Boulle, Robin Wood, Daniela Garone, Jeffrey S. A. Stringer, Timothy B. Hallett, Olivia Keiser, for the IeDEA Southern Africa Collaboration

**Affiliations:** 1 Institute of Social and Preventive Medicine (ISPM), University of Bern, Bern, Switzerland; 2 Centre for Infectious Disease Epidemiology & Research (CIDER), School of Public Health & Family Medicine, University of Cape Town, Cape Town, South Africa; 3 Infectious Diseases Clinic, University Hospital Bern, Bern, Switzerland; 4 Desmond Tutu HIV Centre, Institute for Infectious Disease & Molecular Medicine, University of Cape Town, Cape Town, South Africa; 5 Khayelitsha ART Programme, Médecins Sans Frontières, Cape Town, South Africa; 6 Centre for Infectious Disease Research in Zambia (CIDRZ), Lusaka, Zambia; 7 Department of Obstetrics and Gynecology, University of North Carolina School of Medicine, Chapel Hill, North Carolina, United States of America; 8 Department of Infectious Disease Epidemiology, Imperial College London, London, United Kingdom; Centre de Physique Théorique, France

## Abstract

**Objectives:**

Mortality in patients starting antiretroviral therapy (ART) is higher in Malawi and Zambia than in South Africa. We examined whether different monitoring of ART (viral load [VL] in South Africa and CD4 count in Malawi and Zambia) could explain this mortality difference.

**Design:**

Mathematical modelling study based on data from ART programmes.

**Methods:**

We used a stochastic simulation model to study the effect of VL monitoring on mortality over 5 years. In baseline scenario A all parameters were identical between strategies except for more timely and complete detection of treatment failure with VL monitoring. Additional scenarios introduced delays in switching to second-line ART (scenario B) or higher virologic failure rates (due to worse adherence) when monitoring was based on CD4 counts only (scenario C). Results are presented as relative risks (RR) with 95% prediction intervals and percent of observed mortality difference explained.

**Results:**

RRs comparing VL with CD4 cell count monitoring were 0.94 (0.74–1.03) in scenario A, 0.94 (0.77–1.02) with delayed switching (scenario B) and 0.80 (0.44–1.07) when assuming a 3-times higher rate of failure (scenario C). The observed mortality at 3 years was 10.9% in Malawi and Zambia and 8.6% in South Africa (absolute difference 2.3%). The percentage of the mortality difference explained by VL monitoring ranged from 4% (scenario A) to 32% (scenarios B and C combined, assuming a 3-times higher failure rate). Eleven percent was explained by non-HIV related mortality.

**Conclusions:**

VL monitoring reduces mortality moderately when assuming improved adherence and decreased failure rates.

## Introduction

Since 2003 the number of people receiving antiretroviral therapy (ART) worldwide has increased 16-fold, with 6.6 million people on treatment at the end of 2010 [1]. The scale-up of ART also increased the number of patients experiencing treatment failure, the need for more expensive second-line regimens, and the development of viral resistance [2]. Monitoring of patients starting ART aims to maximize the durability of first-line regimens and to prevent viral resistance. In industrialized countries patients on ART have regular measurements of plasma HIV 1-RNA (viral load, VL) and CD4 cell counts. When drug resistance is suspected, genotypic or phenotypic resistance tests are done. In resource-limited settings monitoring of ART is generally based on CD4 counts or clinical monitoring [3]. The World Health Organization (WHO) has developed clinical and immunologic criteria to detect treatment failure in the absence of VL monitoring [4]. However, the accuracy of these criteria is poor: both sensitivities and positive predictive values of the immunologic and clinical criteria are below 50% [5], [6]. Therefore, many patients are switched late to second line ART, or not switched at all [7].

Studies of the effect of routine VL monitoring on mortality have produced conflicting results. Two randomized trials [8], [9] and one modelling exercise [10] showed that adding VL to CD4 cell count or clinical monitoring did not significantly improve survival. In contrast, another modelling study estimated that viral load monitoring might increase life expectancy by about 10% [11]. We recently compared outcomes between ART programmes in Southern Africa with and without access to VL monitoring [12]. We found that mortality was about 25% higher in the programmes in Malawi and Zambia that monitored CD4 counts only, compared to those from the Republic of South Africa, where VL is also monitored [12].

VL monitoring should lead to more timely detection of treatment failure, more effective, targeted adherence counselling and more appropriate switching to second-line ART, thus reducing exposure to a failing regimen and improving survival. In the present study we used a mathematical model to examine the extent to which the mortality differences observed in patients starting ART in the different settings in Southern Africa [12] might be explained by the use of VL monitoring in some programmes but not others.

## Methods

Our study had two components: first we adapted a previous mathematical model [13] to estimate the causal effect of VL monitoring on mortality from all causes, compared to CD4 cell count monitoring only, under different scenarios. In a second step we examined to what extent VL monitoring could explain the observed difference in mortality between ART programmes in South Africa and Zambia and Malawi observed in a previous analysis [12].

### Antiretroviral Treatment Programmes

The International epidemiologic Databases to Evaluate AIDS in Southern Africa (IeDEA-SA) is a regional collaboration of ART programmes, which is part of a large international network [14]. Data are collected at ART initiation and each follow-up visit, using standardized instruments, and transferred in regular intervals to data centres at the Universities of Cape Town, South Africa, and Bern, Switzerland. All sites have ethical approval to collect data and participate in IeDEA-SA. The previous analysis [12] included four public-sector ART programmes from South Africa, which monitor VL and CD4 cell counts every 3–6 months (Khayelitsha [15], Gugulethu [16] and the Tygerberg clinic [17] in Cape Town, and the Themba Lethu clinic [18] in Johannesburg) as well as the Lighthouse clinic at Kamuzu Central Hospital in Lilongwe [19], Malawi, and the Ministry of Health – Centre for Infectious Disease Research in Zambia (MoH-CIDRZ) programme in Lusaka, Zambia [20]. All six programmes trace patients lost to follow-up (LTFU).

### Definitions

First-line ART was defined as a regimen of two nucleoside reverse transcriptase inhibitors (NRTIs) and one nonnucleoside reverse transcriptase inhibitor (NNRTI). A switch to a second-line regimen was defined as a change from the initial NNRTI-based regimen to a protease inhibitor-based regimen after at least 6 months of follow-up. Virologic treatment failure was defined as a plasma viral load ≥1000 copies/ml. Immunologic failure was defined according to the WHO, as either a CD4 cell count <100 cells/µl, below baseline or a decrease of at least 50% from the on-treatment peak value [13]. In case of suspected failure, an additional measurement (CD4 count or VL, depending on strategy) was taken 3 months later, and if the corresponding failure criteria were met again, the patient was classified as failing treatment at that time. We compared two monitoring strategies: one where decisions to switch to second-line ART were based on CD4 counts and another one where decisions were based on VL monitoring. In both strategies measurements were taken every 6 months.

### Mathematical Model

We adapted a previously developed individual-based mathematical model to simulate outcomes after ART initiation in a cohort of 1000 HIV-infected adult patients [13]. In brief, we simulated progression events for each patient, including immunologic and virologic failure on first-line ART, switching to second-line therapy, immunologic and virologic failure on second-line ART, loss to follow-up (LTFU) and death. Mortality was separated into HIV-related mortality and non-HIV related (background) mortality. Mortality estimates are based on the observed mortality, and virologic and immunologic failures increase the risk of death independently of each other. Due to the high LTFU rate, the observed mortality underestimates the true mortality of patients who started ART [21]. To take this into account, we obtained corrected mortality estimates where the mortality among patients LTFU is estimated based on a systematic review of studies that traced patients LTFU and ascertained their vital status [21], [22]. A more detailed description of the model and approach to dealing with LTFU is given in the Appendix S1.

The model was parameterized using data from the Khayelitsha [15] and Gugulethu [16] ART programmes, the two cohorts with the most complete VL data. Data from the MoH-CIDRZ cohort in Zambia [20] were used to evaluate switching rates among patients without VL monitoring. A detailed description of the dataset including a comparison to the outputs of the model is given in the Appendix S1. The model parameters and their sources are shown in Table 1.

**Table 1:** Model parameters and data sources.

Outcome	Source	Statistical model	Starting	Value (95% CI)	Dimension	Risk
**Time to virologic failure**						
First-line ART; second-line ART withimmediate switch	Cohorts	Parametric Weibull	3 months fromART start	0.47 (0.43–0.50)	Shape	5.6% fail by 1 year after ART start
				3.30 (2.77–3.95)	Scale (100 years)	
Resistance penalty	[11]	*)	n/a	0.05 (0.00–0.20)	Decrease in ART efficacy	n/a
**Time to immunologic failure**						
After virologic failure	Cohorts	Parametric exponential	Virologic failure	0.08 (0.06–0.10)	Rate (years^−1^)	7.6% fail by 1 year after virologic failure
Before virologic failure	Cohorts	Parametric Weibull	3 months fromART start	0.22 (0.20–0.25)	Shape	3.0% fail by 1 year after ART start
				5.46 (3.14–9.51)	Scale (10^6^ years)	
**Time to death and LTFU**						
Non-HIV related mortality, men	ASSA2008 [25]	No specific model**)	Birth	67	Median (years)	21% die by age of 50
Non-HIV related mortality, women	ASSA2008 [25]	No specific model**)	Birth	72	Median (years)	13% die by age of 50
HIV-related observed mortality	Cohorts and ASSA2008 [25] ***)	Double Weibull****)	ART start	0.92 (0.92–0.92)	Shape 1	8.4% have died 1 year after ART start
				0.30 (0.30–0.30)	Scale 1 (years)	
				1.00 (1.00–1.00)	Shape 2	
				124.25 (121.27–127.31)	Scale 2 (years)	
				0.06 (0.06–0.06)	Weight (1^st^ component)	
LTFU	Cohorts	Double Weibull****)	ART start	0.94 (0.94–0.94)	Shape 1	4.2% LTFU 1 year after ART start
				1.00 (1.00–1.00)	Scale 1 (years)	
				25.45(25.45–25.45)	Shape 2	
				66.19(66.19–66.19)	Scale 2 (years)	
				0.07 (0.07–0.07)	Weight (1^st^ component)	
Extra hazard after immunologic failure	Cohorts	Cox regression	Immunologic failure	1.75 (1.15–2.67)	HR, constant over time	n/a
Extra hazard after virologic failure	Cohorts	Cox regression	Virologic failure	1.07 (0.98–1.18)	HR per 3 months on failing ART	n/a
**Observed delay in switching**						
After virologic failure	Cohorts	Parametric exponential	Virologic failure	0.75 (0.63–0.89)	Rate (years^−1^)	53% switched 1 year after virologic failure
After immunologic failure	Cohorts*****)	Parametric exponential	Immunologic failure	0.06 (0.05–0.08)	Rate (years^−1^)	6% switched 1 year after immunologic failure

Distributions of times to event were assumed to be exponential, Weibull or double Weibull, based on the cohort data. Cohort data are from the Khayelitsha and Gugulethu ART programmes in Cape Town, South Africa, unless otherwise specified.

CI, confidence interval; ART, antiretroviral therapy; HR, hazard ratio; ASSA, Actuarial Society of South Africa; LTFU, loss to follow-up; n/a, not applicable.

* )Relative decrease in second-line efficacy per year spent on failing first-line ART.

** )Age-specific mortality rates.

*** )Non-HIV related mortality estimated from the ASSA2008 model deducted from cohort data on all-cause mortality.

**** )Weighted sum of two Weibull distributions.

***** )Data from Ministry of Health-Centre for Infectious Disease Research in Zambia.

### Modelling of the Effect of VL Monitoring on Mortality

In the first analysis, we simulated cohorts with identical baseline characteristics to compare 5-year mortality between VL monitoring and CD4 monitoring. Three scenarios were compared: In baseline scenario A, we assumed that patients switch to second-line ART according to the guidelines, i.e. immediately after meeting the relevant failure criteria. All other parameters including non-HIV related background mortality were also assumed to be identical between the two strategies. Any difference in mortality would thus be due to the ability to detect treatment failure more accurately when using routine VL compared to CD4 monitoring, leading to more timely switching to second-line ART.

In scenario B we investigated the effect of reluctance to switch by introducing a delay from meeting the failure criteria to switching. Sites without access to VL monitoring tend to have lower switching rates than sites with regular VL measurements [7]. The times from confirmed failure to switching were estimated separately from sites with (Gugulethu, Khayelitsha) and without (MoH-CIDRZ) viral load monitoring.

Lower virologic failure rates as a consequence of better adherence in sites with routine VL monitoring could also reduce mortality [23]. In scenario C we therefore increased the risk of the (unobserved) virologic failure in the CD4 monitoring cohort by adjusting the scale parameter of the corresponding Weibull distribution. We investigated the effect of a 2-times and 3-times higher risk of failure with CD4 count compared to VL monitoring. This range of virologic failure rates was observed in a systematic review of cross-sectional studies of virologic failure that included both sites with and without VL monitoring [24].

In all three scenarios baseline characteristics were identical across strategies and age- and sex-specific background mortality rates were for Africans living in the Western Cape in 2007 [25]. We assumed that after second line failure no further treatment options were available. We ran the model 1000 times for both strategies, sampling the parameters for each run from the appropriate distribution. The results from the simulations are presented as relative risks (RR) of death from all causes with 95% prediction intervals (PrI) comparing VL with CD4 cell count monitoring. Estimates of cumulative mortality at 5 years, uncorrected and corrected for LTFU are also given. We used a weighted average approach to correct mortality for LTFU [22] (see Appendix S1 for details).

### Comparison of Model Predictions with Observed Data

In the second analysis, we aimed to determine to what extent VL monitoring could explain the observed difference in mortality between VL (South Africa) and CD4 (Zambia and Malawi) sites [12]. We first did a simulation that reflected the situation in the South African sites, with delays in switching as observed in these cohorts. We then did a series of simulations where the differences between Malawi and Zambia and South Africa were introduced one by one: (i) a 29% higher background mortality [12]; (ii) CD4 monitoring instead of VL monitoring (scenario A); (iii) delay in switching to second-line ART (scenario B); and additionally virologic failure rates (iv) 2-times and (v) 3-times higher with CD4 count than with VL monitoring (scenarios B and C combined). For each simulation, we ran 100 cohort simulations of 1000 patients. We show Kaplan-Meier curves of mortality, modelled mortality estimates at different time points, and report the proportion of the observed difference in mortality between Malawi and Zambia and South Africa that was explained by the three scenarios.

## Results

### Modelled Effect of VL Monitoring on Mortality

#### Scenario A (baseline scenario)

The low accuracy of the WHO immunologic failure criteria meant that only 8% of virologic failures could be detected by CD4 monitoring within one year (Table 1). Mortality at 5 years was nevertheless only slightly lower with VL monitoring compared to CD4 count monitoring: the relative risk was 0.94 (95% PrI 0.74–1.03). Cumulative mortality 5 years after the start of ART was 12.3% (95% PrI 9.8–15.0) with VL monitoring and 13.1% (95% PrI 9.9–19.3) with CD4 monitoring, assuming that patients switch immediately after detection of treatment failure (scenario A in Table 2). Taking into account mortality among patients lost to follow-up increased these estimates to 16.5% (95% PrI 13.6–19.5) with VL monitoring and 17.3% (95% PrI 13.9–22.4) with CD4 count monitoring.

**Table 2:** All-cause mortality after five years on antiretroviral therapy (ART) – 1000 simulations of 1000 patients in cohorts with or without routine viral load monitoring.

	Mortality 5 years after ART start (95% prediction interval)		Risk ratio*** (95% prediction interval)
	Uncorrected*	Corrected**	
**A) Baseline scenario**			
Viral load monitoring	12.3% (9.8–15.0)	16.5% (13.6–19.5)	0.94 (0.74–1.03)
CD4 cell monitoring	13.1% (9.9–19.3)	17.3% (13.9–22.4)	1
**B) Delayed switching**			
Viral load monitoring	12.6% (9.7–16.7)	16.8% (13.5–20.3)	0.94 (0.77–1.02)
CD4 cell monitoring	13.5% (9.7–20.5)	17.6% (13.8–23.7)	1
**C) Higher virologic failure rates with CD4 monitoring**			
Rate of virologic failure 2× higher with CD4 monitoring compared to viral load monitoring:			
Viral load monitoring	12.3% (9.8–15.0)	16.5% (13.6–19.5)	0.86 (0.54–1.05)
CD4 cell monitoring	14.2% (9.1–27.0)	18.3% (13.5–29.5)	1
Rate of virologic failure 3× higher with CD4 monitoring compared to viral load monitoring:			
Viral load monitoring	12.3% (9.8–15.0)	16.5% (13.6–19.5)	0.80 (0.44–1.07)
CD4 cell monitoring	15.4% (9.2–33.5)	19.4% (13.6–35.5)	1

ART, antiretroviral therapy; VL, routine viral load monitoring.

A (baseline scenario): identical virologic failure rates in both monitoring strategies, switch to second-line ART immediately after confirmed failure. B (delayed switching): identical virologic failure rates in both monitoring strategies, switch to second-line ART after a realistic delay (see Table 1 for parameters). C (higher virologic failure rates with CD4 monitoring): rate of virologic failure set to be 2 or 3 times higher with CD4 monitoring by adjusting the scale parameter of the Weibull distribution (Table 1), switch to second-line ART immediately after confirmed failure.

* Uncorrected mortality: mortality based on observed mortality from data.

** Corrected mortality: mortality based on observed mortality, observed LTFU and estimated mortality among patients lost [22].

*** Ratios of uncorrected mortality, comparing VL with CD4 monitoring.

#### Scenario B (delayed switching)

The proportion of patients who switched within one year of meeting relevant failure criteria was 53% in programmes with routine VL monitoring and 6% among patients with CD count monitoring (Table 1). Again, mortality at 5 years was only slightly reduced with VL monitoring: the RR was 0.94 (95% PrI 0.77–1.02) and cumulative mortality was 12.6% (9.7–16.7) with routine VL monitoring and 13.5% (9.7–20.5) with CD4 count monitoring. The corresponding estimates corrected for loss to follow-up were 16.8% (13.5–20.3) and 17.6% (13.8–23.7) (scenario B in Table 2).

#### Scenario C (increased rates of virologic failure)

Assuming a higher rate of virologic failure in the CD4 monitoring cohort had a more substantial impact (Scenario C in Table 2): the relative risks comparing VL with CD4 monitoring were 0.86 (0.54–1.05) for a 2-times higher rate and 0.80 (0.44–1.07) for a 3-times higher rate of virologic failure. The corresponding estimates of corrected mortality in the CD4 cohort at 5 years were 18.3% (13.5–29.5) and 19.4% (13.6–35.5), respectively.

### Comparisons of Model Predictions with Observed Mortality

Observed mortality was based on 18 706 adult patients starting ART in South Africa and 80 937 patients starting ART in Zambia or Malawi [12]. Patients from viral load sites were more likely to be women (66% vs. 62%) and had lower CD4 cell counts (93 versus 132 cells/µl) at the start of therapy. In both settings, most patients started ART with a regimen that combined lamivudine and stavudine (3TC/d4T) either with nevirapine or efavirenz. Zidovudine (ZDV), didanosine (ddI) and boosted lopinavir (LPV/r) was the most common second-line regimen in South Africa, whereas in Malawi and Zambia, a combination of tenofovir (TDF), emtricitabine (FTC) and LPV/r was most commonly used.

**Figure 1:**
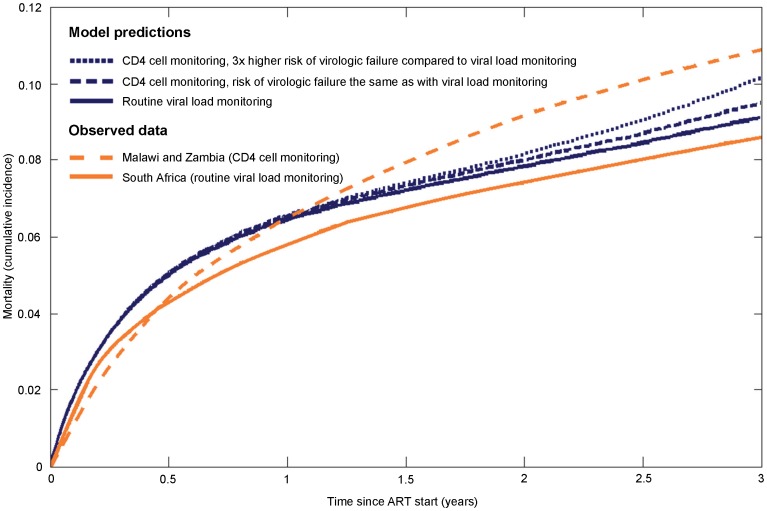
Comparison of all-cause mortality based on model predictions and observed data. Orange lines show Kaplan-Meier estimates from ART programmes in South Africa, Malawi and Zambia [12] and blue lines the model predictions. Solid lines represent routine viral load monitoring (South Africa) and broken lines CD4 cell monitoring (Malawi, Zambia).

Modelled mortality and Kaplan-Meier estimates of observed mortality in the South African VL programmes were 9.1% and 8.6% at 3 years of ART, respectively. In Malawi and Zambia, with CD4 monitoring only, the corresponding modelled estimates ranged from 9.5% (scenarios A and B) to 10.1% (scenario B combined with C, assuming a 3-times higher virologic failure rate). The Kaplan-Meier estimate of mortality at 3 years in the CD4 monitoring only cohorts was 10.9% (Figure 1). During the first 1.5 years on ART, the modelled mortality was higher than the observed morality, and little difference was seen between the three scenarios. After 1.5 years differences in mortality between modelled scenarios increased gradually.

**Figure 2:**
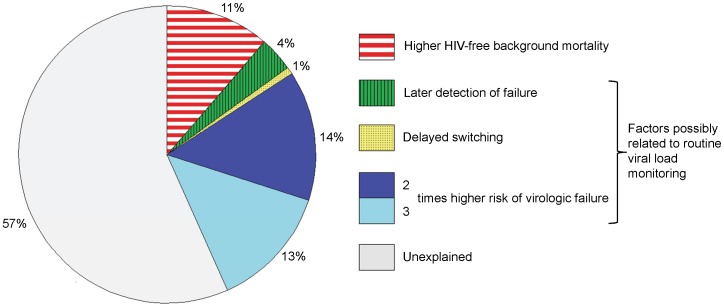
Possible explanations for the difference in mortality at three years of antiretroviral therapy between South Africa and Malawi and Zambia. The graph shows the proportion that different causes may contribute to the higher mortality observed in Malawi and Zambia (CD4 cell count monitoring) compared to South Africa (VL monitoring). The estimates are based on the mathematical model. The effect of a higher risk of virologic failure in sites with CD4 count monitoring is shown for a 2-times higher risk (dark blue) and 3-times higher risk (light and dark blue combined).

The absolute difference in observed mortality between Malawi and Zambia and South Africa was 2.3% (10.9%–8.6%). Approximately 4% of the observed difference in mortality could be explained by more complete detection of virologic failure with VL monitoring (Figure 2). The delay from failure to switching explained only 1% of the difference. When we assumed that VL monitoring decreased rates of virologic failure (through improved adherence) the percentage of the mortality difference explained by viral load monitoring increased to 19% (assuming a 2-times higher failure rate) or 32% (3-times higher failure rate). Differences in HIV-unrelated background mortality explained 11% of the observed difference (Figure 2).

## Discussion

We used a mathematical model to estimate the causal effect of VL monitoring, compared to CD4 count monitoring, on mortality in ART programmes and to examine to what extent the higher mortality among patients starting ART in Zambia and Malawi, compared to South Africa [12], might be explained by the routine monitoring of VL in South Africa but not in the other two countries. We found that the relative reduction in mortality associated with VL monitoring was small (6%) when assuming that VL monitoring exclusively led to more timely and complete detection of treatment failure but more substantial (20%) when also assuming that VL monitoring reduced the rate of virologic failure. Under the latter scenario up to a third of the absolute difference in mortality observed between South Africa and Malawi and Zambia could be attributed to VL monitoring in South Africa. Possible reasons for the remaining difference include the more effective management of opportunistic infections and cancers, including access to intensive care in South Africa [12], differences in ascertainment of deaths between cohorts, differences in mortality due to different rates of loss to follow-up and differences in baseline characteristics.

The results of previous modelling studies of the VL monitoring and mortality have been heterogeneous. Phillips *et al* found that viral load monitoring increased the average survival time in the first 5 years of ART from 4.09 to 4.14 [10]. This corresponds to a RR of death comparing VL with CD4 count monitoring of about 0.95 at 5 years. Kimmel et al reported that VL monitoring increased the life expectancy by 10% as compared to CD4 monitoring. This corresponds to a RR of 0.92 [11]. Our estimated relative risk of 0.94 is thus in line with the previous studies. However, we also show that the impact of VL monitoring could be substantially greater if other benefits such as the reduction in the risk of virologic failure are considered.

Despite the fact that poor adherence is a major predictor of virologic failure [26], the effect of VL monitoring on adherence rates has not been considered in previous modelling studies. In a large treatment programme in South Africa, the majority of patients with VL rebounds above 100 copies/ml re-suppressed viral replication to undetectable levels after a targeted adherence intervention [23]. As a result, only 2% of patients had virologic failure after one year of follow-up. We found higher failure rates with about 6% of patients experiencing virologic failure at one year, 8% at two years and almost 10% at three years. A systematic review of ART programmes in sub-Saharan Africa found that the median percentage of patients experiencing virologic failure (>1000 copies/ml) was 14% at 3 to 48 months of follow-up [24]. In a cross-sectional study from Cameroon, where patients had to pay out of pocket for VL measurements, 16% had a VL >1000 copies/ml after one year and 23% after 2 years on ART [27]. Unfortunately different definitions and follow-up durations hamper more detailed comparisons of rates of virologic failure. In our model we assumed an up to 3-times higher virologic failure rate in the absence of VL monitoring. This assumption may seem strong but it is consistent with the literature.

The effect of VL monitoring on mortality and other outcomes should ideally be examined in adequately powered randomized controlled trials. For ethical reasons the protocols of randomized trials will tend to resemble scenario A in our modelling study, where trial participants are monitored closely for adherence and clinical symptoms and thorough adherence counselling is implemented in all groups. Indeed, in the only trial comparing VL with CD4 count monitoring published so far, all patients were visited weekly by a trained lay person using a standardized symptoms questionnaire. Patients were weighed each month and drugs were replaced weekly using pre-packed storage containers [8]. This Ugandan trial found no clear benefit of routine VL monitoring on mortality: the hazard ratio comparing VL with CD4 count monitoring was 0.93, which is approximately the same as in the present study when modelling scenario A. Of note, the trial was not powered to detect or exclude smaller differences in mortality and confidence intervals around the hazard ratio were wide (0.59 to 1.45) [8].

A non-inferiority trial in rural district hospitals in Cameroon [9] that compared clinical and laboratory monitoring (VL and CD4 cell counts every 6 months) with clinical monitoring alone confirmed the reluctance to switch in the absence of documented virologic failure observed in cohort studies [7] and modelled in our study: 13 of 237 patients (6%) in the laboratory group switched to second-line ART compared to none in the clinical monitoring group [9]. This is not surprising: when switching patients based on clinical or immunologic failure criteria many will switch unnecessarily (i.e. with undetectable VL) [5], [28], [29]. Second-line therapy is much more expensive [30], the pill burden generally higher [4], adverse effects more frequent [31] and second-line ART is the last treatment option in many settings. Our model indicates that such reluctance to switch is not, however, associated with substantially increased mortality.

The beneficial effect of VL monitoring on adherence may thus be masked in randomized trials due to intensive counselling and clinical monitoring in all arms. Furthermore, patients who participate in clinical trials can generally be expected to be more adherent than patients treated in routine ART programmes. Although there are at least two ongoing trials that compare routine VL to CD4 monitoring [32]–[33], it is unclear to what extent these studies will reflect routine care in programmes with and without access to VL monitoring. More data on levels of adherence and the rate of virologic failure are thus needed both from routine programmes and trials.

Our study had several limitations. First, the probability of HIV-related outcomes in our simulations depended on only two patient-level factors – virologic and immunologic treatment response. We thus ignored factors such as age and gender of the patient, adherence to treatment, resistance mutations, baseline CD4 and VL values, opportunistic illnesses and co-infections. Although some of these factors were recorded, we decided to keep the structure of the model simple. We stress that our results reflect the situation of the ART programmes included in this study, and may not apply to other settings. Nevertheless, these cohorts are typical for adult ART programmes in Southern Africa, with the majority of patients being women and most patients starting ART with low CD4 cell counts. Second, we assumed a constant rate of immunologic failure following virologic failure: we did not have sufficient data to estimate the progression to immunologic failure more precisely. Third, we assumed that the hazard of death after virologic failure increased over time whereas the hazard after immunologic failure remained constant, in line with previous studies [34]. In the comparison of model predictions with observed mortality we did not take differences in baseline characteristics between the cohorts in South Africa and Malawi and Zambia into account but examined the crude absolute difference in mortality observed between the two settings. CD4 cell counts were lower in the VL sites in South Africa and it is therefore likely that adjustment for baseline characteristics would have increased this difference, and reduced the proportion explained by VL monitoring of ART in South Africa.

We restricted our simulations to two strategies and five years of follow-up, and did not include costs or the effect on HIV transmission. Additional strategies could include CD4 monitoring with targeted VL monitoring (i.e. in patients experiencing immunologic failure), CD4 or VL monitoring with different measurement frequencies, or VL monitoring with different failure thresholds. A recent study confirmed the key role of targeted VL monitoring to prevent unnecessary switching and to reduce costs [28] but its impact on survival is probably small. Targeted VL monitoring will increase switching among virologically failing patients, which should improve their survival, but the majority of failing patients would go on undetected. Although the individual benefits of VL monitoring may be modest, routine VL may have more substantial benefits in the prevention of new infections and by limiting the spread of drug resistance. We previously found that VL monitoring may prevent about 30% of transmission from treated patients [13].

In conclusion, we have shown that routine VL monitoring can reduce mortality over the first five years of ART. The magnitude of this benefit depends on the ability of VL monitoring to improve adherence and therefore decrease rates of virologic failure. As point-of-care VL testing will be introduced in programmes in resource-limited settings in the near future, more research is urgently needed to improve our understanding of how VL monitoring impacts on disease progression, the development of drug resistance and HIV transmission at the individual and population level.

## Supporting Information

Figure S1 Schematic representation of disease progression on ART in the mathematical model.
(DOCX)

Figure S2 Comparison of the main outcomes between the mathematical model and the source data.
(DOCX)

Table S1 Patient characteristics at start of antiretroviral therapy in the dataset that was used to parameterise the mathematical model.
(DOCX)

Appendix S1 Description of the mathematical model and data sources.
(DOCX)
