# Supplementary material for: Monitoring of Antiretroviral Therapy and Mortality in HIV Programmes in Malawi, South Africa and Zambia: Mathematical Modelling Study
Source: PLoS One. 2013 Feb 28;8(2):e57611. doi: 10.1371/journal.pone.0057611 (PMC3585414; doi:10.1371/journal.pone.0057611)
Supplement: Figure S1 — Schematic representation of disease progression on ART in the mathematical model. (DOCX) [file pone.0057611.s001.docx]

**Figure S1. Schematic representation of disease progression on ART in the mathematical model.** Solid arrows represent natural disease progression, dashed arrows switching with viral load monitoring and dotted arrows switching with CD4 monitoring. Immunologic failure can be caused either by virologic failure (*) or other causes (**). The patient can also proceed to death or LTFU from any of the shown stages**.**

1^st^-line ART with virologic and immunologic** failure

Successful 2^nd^-line ART

2^nd^-line ART with virologic failure

2^nd^-line ART with immunologic** failure

2^nd^-line ART with virologic and immunologic* failure

2^nd^-line ART with virologic and immunologic** failure

1^st^-line ART with virologic and immunologic* failure

Successful 1^st^-line ART

1^st^-line ART with virologic failure

1^st^-line ART with immunologic** failure
