# Supplementary material for: Monitoring of Antiretroviral Therapy and Mortality in HIV Programmes in Malawi, South Africa and Zambia: Mathematical Modelling Study
Source: PLoS One. 2013 Feb 28;8(2):e57611. doi: 10.1371/journal.pone.0057611 (PMC3585414; doi:10.1371/journal.pone.0057611)

**Figure S2. Comparison of the main outcomes between the mathematical model and the source data.** The model was run 100 times and parameters were sampled from distributions using Latin Hypercube sampling. The grey solid lines show the cumulative incidence of each model run separately, the black solid line the overall cumulative incidence of all model runs combined, and the dashed curve the cumulative incidence in the observed dataset that was used to parameterize the model.


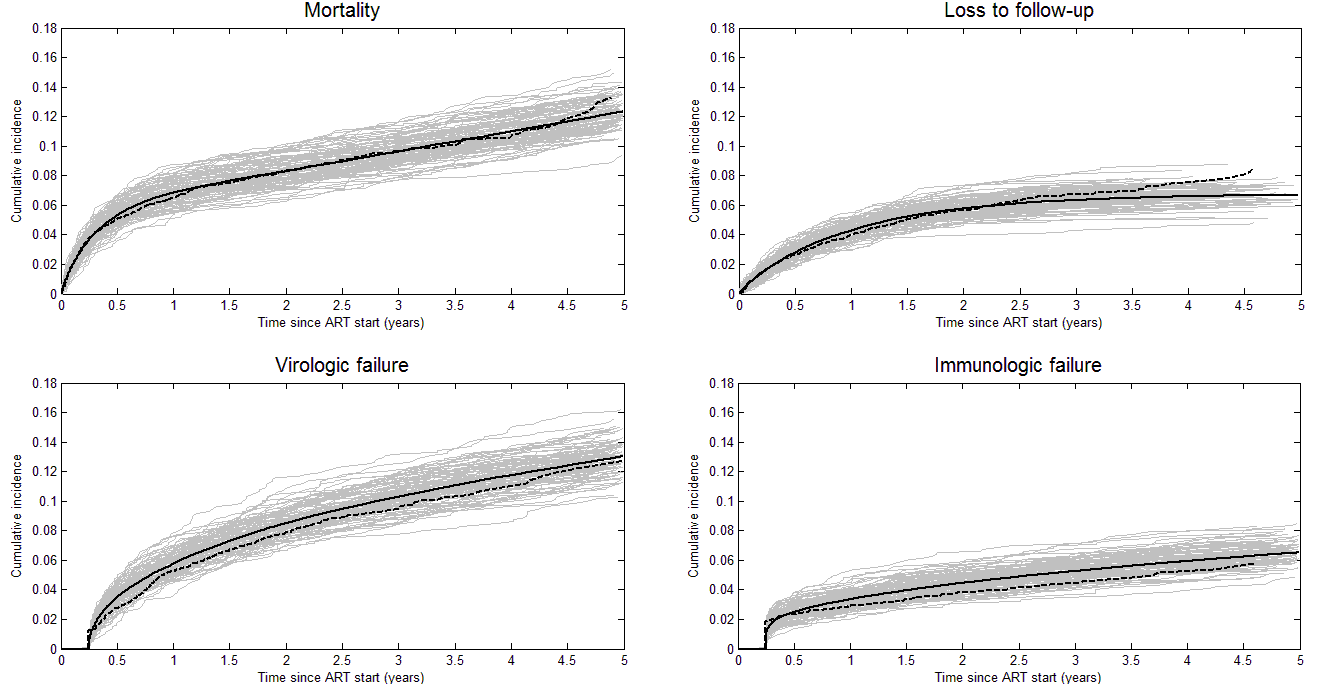

Supplement: Figure S2 — Comparison of the main outcomes between the mathematical model and the source data. (DOCX) [file pone.0057611.s002.docx]
