# Supplementary material for: Monitoring of Antiretroviral Therapy and Mortality in HIV Programmes in Malawi, South Africa and Zambia: Mathematical Modelling Study
Source: PLoS One. 2013 Feb 28;8(2):e57611. doi: 10.1371/journal.pone.0057611 (PMC3585414; doi:10.1371/journal.pone.0057611)
Supplement: Table S1 — Patient characteristics at start of antiretroviral therapy in the dataset that was used to parameterise the mathematical model. (DOCX) [file pone.0057611.s003.docx]

**Table S1. Patient characteristics at start of antiretroviral therapy in the dataset that was used to parameterise the mathematical model.**

|  | **Analysis of mortality and loss to follow-up (n=9888)** |
| --- | --- |
| **Age (years)** |  |
| Median (IQR) | 33 (29-39) |
| **Gender** |  |
| Male | 3240 (32.8%) |
| Female | 6648 (67.2%) |
| **Cohort** |  |
| Gugulethu | 2658 (26.9%) |
| Khayelitsha | 7230 (73.1%) |
| **Baseline CD4 cell count (cells/μL)** | |
| Median (IQR) | 93 (41-158) |
| **Baseline HIV-1 viral load (log10 copies/mL)** | |
| Median (IQR) | 5.0 (4.5-5.5) |
| **First-line regimens** |  |
| d4T 3TC EFV | 4985 (50.4%) |
| d4T 3TC NVP | 3182 (32.2%) |
| ZDV 3TC NVP | 1031 (10.4%) |
| ZDV 3TC EFV | 680 (6.9%) |
| d4T ddI EFV | 7 (0.1%) |
| ZDV ddI EFV | 3 (0.0%) |
| **Second-line regimens** |  |
| ZDV ddI LPV | 243 (69.2%) |
| ZDV 3TC EFV LPV | 33 (9.4%) |
| d4T ddI LPV | 22 (6.3%) |
| ZDV 3TC LPV | 9 (2.6%) |
| Other | 44 (12.5%) |

IQR, interquartile range; 3TC, lamivudine; d4T, stavudine; ZDV, zidovudine; ddI, didanosine; NVP, nevirapine; EFV, efavirenz; LPV, lopinavir.
