# Supplementary material for: Monitoring of Antiretroviral Therapy and Mortality in HIV Programmes in Malawi, South Africa and Zambia: Mathematical Modelling Study
Source: PLoS One. 2013 Feb 28;8(2):e57611. doi: 10.1371/journal.pone.0057611 (PMC3585414; doi:10.1371/journal.pone.0057611)
Supplement: Appendix S1 — Description of the mathematical model and data sources. (DOCX) [file pone.0057611.s004.docx]

**Appendix S1**

The mathematical model

A detailed description of the mathematical model has been given in a previous publication [[1](#_ENREF_1)]. In brief, the model is a multistate disease progression simulation model, which defines for each patient a series of events that determine the patient’s progression on ART. Hazard functions that define the timing of each event can be of arbitrary form and take into account previous events. Therefore, the model is non-Markovian. The simulated events include first-line virologic failure, first-line immunologic failure, switch to second-line ART, second-line virologic failure, second-line immunologic failure, death and loss to follow-up (LTFU). A schematic representation of the model is shown in supplementary Figure S1.

Immunologic failure can result from either virologic failure or other causes: these two events are competing and simulated independently of each other. If the immunologic failure is caused by virologic failure, the patient returns to successful ART after switching, but if it is due to other causes, the immunologic failure remains effective and increases mortality also after switching. The times from 3 months after start of ART or switching to virologic failure and non-viral load related immunologic failure were assumed to be Weibull distributed. For the progression from virologic to viral load-related immunologic failure, we assumed a constant rate. In addition, the time from switching to second-line virologic failure was reduced by a resistance penalty factor, which was assumed to be proportional to the time spent on a failing first-line therapy.

At each stage, the patient was at risk of death and LTFU. Mortality was estimated based on the observed deaths and separated into HIV-related mortality and background mortality. LTFU includes unascertained deaths, transfers-out and stopping ART. Both HIV-related mortality and loss to follow-up were fitted with a double Weibull distribution (weighted sum of two Weibull distributions). We assumed that virologic and immunologic failures would increase the HIV-related mortality independently of each other. The hazard ratio related to virologic failure was assumed to increase over time, whereas the hazard ratio related to immunologic failure remained constant after failure.

By including LTFU as a separate outcome we assumed that the mortality which was estimated from the model represents the observed mortality. In reality, mortality among patients lost to follow-up is high and therefore the observed mortality rates probably underestimate true mortality [[2](#_ENREF_2)]. To take this into account, we show also corrected mortality estimates including the estimated mortality among patients lost to follow-up. To calculate the corrected mortality we used a relation between the proportion of patients lost to follow-up *r* and mortality among patients lost to follow-up *M_L_* from a systematic review where patients lost to follow-up were traced [[2](#_ENREF_2), [3](#_ENREF_3)]:

$$M_{L}\left( t \right)=\frac{e^{a+br\left( t \right)}}{1+e^{a+br\left( t \right)}}$$

where *a* = 0.57287 and *b* = -4.04409.

For each model run, we sampled a set of parameters from the distributions given in Table 1 of the main text. We assumed that the following transformations of exponential parameters were normally distributed:

- log *k_i_*
- -*k_i_* log *λ_i_*
- *w* (restricted to [0,1])

where *k_i_*, *λ_i_* and *w* are the shape, scale and weight parameters of a double Weibull distribution (exponential and Weibull distributions are treated as special cases of the double Weibull distribution). A detailed description of the distributions and parameterization can be found in the web appendix of Estill *et al* [[1](#_ENREF_1)].

Data sources

The IeDEA-SA database currently includes over 300,000 patients receiving ART in 19 ART programmes in seven Southern African countries and includes cohorts with and without routine viral load monitoring. IeDEA-SA is part of IeDEA, which includes similar networks in other regions of Africa, America and Asia ([[4](#_ENREF_4)]; [www.iedea.org](http://www.iedea-hiv.org)).

We selected two cohorts from South Africa, Gugulethu and Khayelitsha, which have both viral load and CD4 values available on average at least once a year for at least 50% of the patients. Additionally, data from patients in the Centre for Infectious Disease Research in Zambia (CIDRZ) programme, who experienced immunologic failure and did not have a viral load measurement available to confirm failure, were analyzed to estimate the time from failure to switching in the absence of viral load measurements. The data used to parameterize the model – including the distribution of patient characteristics at start of ART – were taken from the IeDEA-SA cohorts whenever possible. When no data were available, we used data from the literature or made various assumptions which were tested in sensitivity analyses. We also calculated mortality rates from all three programmes and additionally loss to follow-up, virologic failure and immunologic failure rates from Gugulethu and Khayelitsha only to compare the fit of the model to the data.

Inclusion criteria and definitions have been previously described [[5](#_ENREF_5)]: Briefly, we included all treatment naïve patients aged 16 years and older who started ART with a non-nucleoside reverse transcriptase inhibitor (NNRTI)-based regimen and who had at least one day of follow-up. Following the WHO criteria in place at the time of data collection and analysis [[6](#_ENREF_6)] we defined immunologic failure as any of: a decline in the CD4 cell count to the baseline value or below, a decline of at least 50% from the peak value on treatment or a persistent CD4 cell count below 100 cells/µl after six months of antiretroviral therapy. Virologic failure was defined as a plasma HIV viral load value above 1,000 copies/ml. For both immunologic and virologic failure we required two consecutive values within 12 months of each other meeting the criteria. A switch to a second-line ART regimen was defined as a change from the initial regimen to a protease inhibitor (PI)-based regimen after at least six months of follow-up with a simultaneous change in at least one nucleoside reverse transcriptase inhibitor (NRTI).

Most parameters of the model were taken from analyses of the Gugulethu and Khayelitsha data which we had conducted and published previously for another modelling study [[1](#_ENREF_1)]. The baseline characteristics of the patients in this dataset are given in supplementary Table S1. In addition we analysed data on 592 patients in Gugulethu and Khayelitsha who experienced virologic failure and 1,204 patients in CIDRZ who experienced immunologic failure and did not have a viral load measurement available to estimate the delay from meeting the relevant failure criteria until switching to second line ART. We found that in Gugluethu and Khayelitsha, 53% of patients had switched one year after virologic failure, but that in CIDRZ only 6% of patients switched within one year after meeting the immunologic failure criteria.

Comparison of the model outcomes and data

To illustrate the fit of the model to the data, we run the model 100 times with six-monthly routine viral load monitoring and delayed switching, corresponding to the monitoring strategy of the cohorts. We plotted the cumulative incidence of the following outcomes and the data analyses: observed (uncorrected) mortality, LTFU, virologic failure, immunologic failure and switching over time (supplementary Figure S2). Modelled mortality and LTFU fitted the data well. Virologic failure rates were slightly overestimated by the model, which also led to a slight overestimation of the immunologic failure rates. However, since in the data many patients switched before meeting the failure definition, the failure rates of the model and data are not directly comparable.

References

1. Estill J, Aubrière C, Egger M, Johnson L, Wood R, et al. (2012) Viral load monitoring of antiretroviral therapy, cohort viral load and HIV transmission in Southern Africa: A mathematical modelling analysis. AIDS 26: 1403-1413.

2. Brinkhof MW, Pujades-Rodriguez M, Egger M (2009) Mortality of patients lost to follow-up in antiretroviral treatment programmes in resource-limited settings: systematic review and meta-analysis. PLoS One 4: e5790.

3. Egger M, Spycher BD, Sidle J, Weigel R, Geng E, et al. (2011) Correcting mortality for loss to follow up: A nomogram applied to antiretroviral treatment programmes in sub-Saharan Africa. PLoS Med 8: e1000390.

4. Egger M, Ekouevi DK, Williams C, Lyamuya RE, Mukumbi H, et al*.* (2012) Cohort Profile: The international epidemiological databases to evaluate AIDS (IeDEA) in sub-Saharan Africa. Int J Epidemiol 41: 1256-1264.

5. Keiser O, Chi BH, Gsponer T, Boulle A, Orrell C, et al. (2011) Outcomes of antiretroviral treatment in programmes with and without routine viral load monitoring in Southern Africa. AIDS 25: 1761-1769.

6. World Health Organisation (2006) Antiretroviral therapy for HIV Infection in adults and adolescents in resource-limited settings: towards universal access. Recommendations for a public health approach. Available: <http://www.who.int/hiv/pub/guidelines/artadultguidelines.pdf>. Accessed 28 January 2013.
